# Subsite variation of HPV-related p16-expression in oropharynx cancer: Incidence and prognostic impact in a population-based DAHANCA cohort 1986–2020

**DOI:** 10.2340/1651-226X.2025.44027

**Published:** 2025-08-14

**Authors:** Pernille Lassen, Jan Alsner, Hanne Primdahl, Christina Caroline Plaschke, Christian Maare, Jørgen Johansen, Maria Andersen, Mohamad Farhadi, Jens Overgaard

**Affiliations:** a Department of Experimental Clinical Oncology, Aarhus University Hospital, Aarhus, Denmark; b Department of Oncology, Aarhus University Hospital, Aarhus, Denmark; c Department of ENT, Copenhagen University Hospital Rigshospitalet, Copenhagen, Denmark; d Department of Oncology, Herlev Hospital, Copenhagen University, Herlev, Denmark; e Department of Oncology, Odense University Hospital, Odense, Denmark; f Department of Oncology, Aalborg University Hospital, Aalborg, Denmark; g Department of Oncology, Næstved Hospital, Næstved, Denmark

**Keywords:** Oropharyngeal neoplasms, incidence, papillomavirus infections, neoplasms staging, prognosis, radiation oncology

## Abstract

**Background and purpose:**

Separate staging criteria based on human papillomavirus (HPV)-related p16-expression are implemented in the TNM8 classification of oropharyngeal cancer (OPSCC). Based on a nationwide cohort, we provide a detailed description of subsite variation in the age-standardised incidence-rates of OPSCC alongside an evaluation of the prognostic impact of p16-expression according to subsite after primary radiotherapy (RT).

**Patient/material and methods:**

A total of 8,462 Danish OPSCC patients from 1986 to 2020 were identified in the DAHANCA-database, and tumours were grouped into ‘tonsil/base of tongue (BOT)’, ‘neighbouring subsites’ and ‘distant subsites’. Subsite-specific age-standardised incidence-rates were calculated, and outcome-analysis (loco-regional control, disease-free survival and overall-survival 5 years after the completion of RT) stratified by p16-status/subsite and restricted to curatively treated patients only (*N* = 3,387) was performed.

**Results:**

A 5-fold increase in the age-standardised incidence of OPSCC was observed and could be ascribed to the rise in p16-positive tumours of tonsil/BOT and neighbouring subsites only as neither the incidence rates nor the proportion of p16-positivity in distant subsites tumours changed over time. The prognostic impact of p16-status for all endpoints differed significantly across tumour subsites with the strongest association found in tonsil/BOT tumours, a diminishing but still significant impact in neighbouring subsite tumours and no significant impact in tumours arising in distant subsites.

**Interpretation:**

Our findings suggest that grouping all p16-positive OPSCC as one entity for staging and prognostication, as currently done in TNM8, is too simple as it does not accurately depict the differences in tumour biology and the consequent treatment response.

## Introduction

Prolonged exposure to carcinogens from tobacco and excess alcohol consumption remains the primary risk factor for the development of head and neck squamous celle carcinoma (HNSCC) [1], but since the turn of the 21st century, it has been evident that infection with high-risk human papillomavirus (HPV) is aetiologically linked to a subgroup of the tumours [2, 3]. Oncogenetic HPV has been detected in HNSCCs from all subsites, but the strongest association is found in oropharyngeal squamous cell carcinoma (OPSCC) [4, 5], where carcinomas of the tonsils are particularly associated with HPV infection [6, 7]. Initial investigations of the potential impact of the virus on treatment outcome included all HNSCC subsites [8–11], but soon, the scope narrowed in to focus on OPSCC only, as there appear to be no prognostic impact of HPV in tumours arising outside oropharynx [12]. In OPSCC, on the other hand, it is clear that the virus profoundly influences the incidence, treatment sensitivity and prognosis for patients with HPV-related disease compared to the HPV-negative counterparts.

In light of the distinct clinical behaviour and favourable prognosis of HPV-related OPSCC compared to HPV-negative disease [13, 14], separate staging criteria of OPSCC based on tumour p16-status were implemented in the 8th edition of the Union for International Cancer Control/American Joint Committee on Cancer TNM classification (TNM8) [15]. However, grouping all OPSCC subsites as one entity may prove to be too rigid, and recent data indicate significant oropharyngeal subsite differences in both the frequency of HPV-relation and in the survival probability of the patients [16, 17]. Whilst the viral association and prognostic implication in tumours arising in the tonsil and base of tongue (BOT) are indisputable, the relation seems less robust in other oropharyngeal subsites where the connection to lymphoepithelial tissue is less obvious or even non-existing. Moreover, as these tumours are also much less prevalent than tonsil/BOT OPSCC, they are typically grouped as one entity (‘other OPSCC’), which inevitably entails the risk of covering potentially more nuanced subsite differences in this regard. Well-conducted, population-based investigations with long-term follow-up, standardised diagnostic criteria and uniform national treatment strategies have the potential of shedding light not only to geographical differences in HPV-related OPSCC but also on these oropharyngeal subsite differences regarding the incidence of the tumours, the frequency of HPV-related p16-expression as well as the prognostic impact. Based on this nationwide, population-based study with prospectively collected data on consecutive OPSCC patients collected over 35 years, we provide a detailed description of the age-standardised incidence rates of OPSCC according to the oropharyngeal subsite. Furthermore, we investigate potential subsite variation regarding the prognostic impact of p16-expression on outcome in patients treated with curatively intended (chemo)radiotherapy.

## Patients/material and methods

### Study population

The Danish Head and Neck Cancer Group (DAHANCA) has registered information about patients diagnosed with HNSCC in the national DAHANCA-database since 1971 [18]. At least annually, a specific comparison to the Danish Cancer Registry is performed to ensure mutual completeness in the coverage of patients, and consequently, the DAHANCA-database consistently maintains nationwide coverage of Danish HNSCC patients.

**Figure 1:**
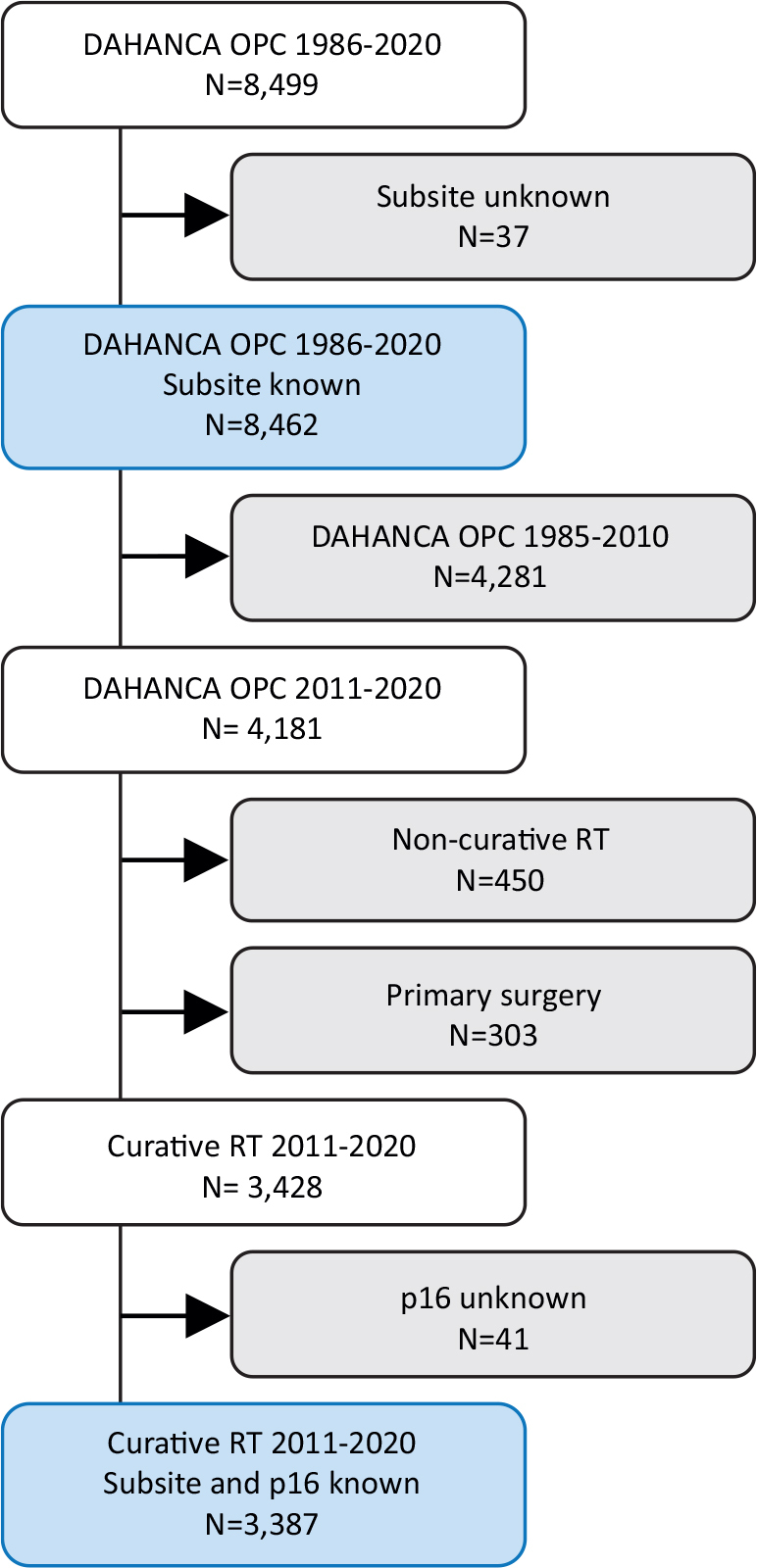
Cohort derivation of OPSCC treated with curative intent, with the two blue boxes corresponding to the 1986–2020 cohort in Figure 2 and the 2011–2020 cohort in Figure 3.

For the present incidence analysis, all patients living in Denmark at the time of their diagnosis and referred to the treatment of histopathological proven stage I–IV OPSCC diagnosed between 1986 and 2020 were included in order to describe the age-standardised incidence rates and frequency of HPV-related p16-status according to the oropharyngeal subsite. Tumours with unknown subsite specification were excluded (*N* = 37), yielding a total cohort of 8,462 patients, Figure 1: upper blue box.

Outcome analysis was restricted to include patients treated with curatively intended primary (chemo)radiotherapy, known p16-status and subsite specification, in the 10-year period from 2011 to 2020, Figure 1: lower blue box. Treatment was done according to the national DAHANCA guidelines [19] using moderately accelerated fractioned radiotherapy (ART) with contemporary technique (intensity-modulated radiotherapy, IMRT), hypoxic modification and concurrent weekly low-dose cisplatin (ART+C) for eligible patients with advanced disease stage (T3/T4 and/or N+).

HPV-association was determined by p16 immunohistochemistry (IHC) staining, and tumours were classified as p16-positive in case of strong and diffuse nuclear or cytoplasmatic staining in > 70% of tumour cells [20, 21]. Smoking-status and smoking-history were prospectively recorded as previously described [14, 22].

Oropharyngeal subsite specification is registered in the DAHANCA-database by the surgeon performing the diagnostic biopsy according to the anatomical areas defined in the TNM classification. Tumours were subsequently grouped based on the proximity/distance to the lymphoepithelial tissue into tumours of the tonsil/BOT, ‘neighbouring subsites’ (tonsillar fossa & arch of the palate, glossotonsilar sulci and vallecula) and ‘distant subsites’ (inferior surface of the soft palate, posterior wall and uvula).

The TNM8 classification was implemented by the DAHANCA in 2018, and before that the TNM6 (2008–2012) and TNM7 (2013–2017) were used. In order to capture these changes in classification over time in a pragmatic way, for the present study, all tumours were reclassified according to a ‘simplified’ disease stage I-II-III and IV model, Supplementary Table 1.

### Statistics

Age-standardised incidence rates, standardised to the 2020 Danish population age distribution, according to the oropharyngeal subsite from 1986 to 2020 is presented in 5-year diagnosis periods (1986–1990, 1991–1995, 1996–2000, 2001–2005, 2006–2010, 2011–2015 and 2016–2020). The frequency of p16-expression by subsite was calculated and displayed for the same 5-year periods.

Features of p16-positive and p16-negative tumours were compared using the Chi^2^ test for categoric variables. For outcome analysis, the end points used were 5-year loco-regional control (LRC), disease-free survival (DFS) and overall survival (OS). LRC was defined as complete and persistent disappearance of the disease in the primary tumour and regional lymph nodes after radiotherapy. Failure was recorded in the event of recurrent tumour or if the primary tumour never completely disappeared. The end point does not, therefore, include the effect of a successful procedure with salvage surgery. Vital status (dead/alive) was checked by linkage to the Danish Civil Registration Service on June 1st 2024. For DFS, events were loco-regional failure, distant metastasis or death from any cause. For OS, events were death from any cause. Outcomes were measured from the first contact with the oncology centre to either death, migration, time of assessment (June 2024) or 5 years after diagnosis, whichever came first. Outcomes were truncated at 5 years, and later events were censored at that point. DFS and OS were calculated and illustrated using the Kaplan-Meier estimator, and the median follow-up time was calculated using the reverse Kaplan-Meier method.

## Results

### Incidence

**Figure 2:**
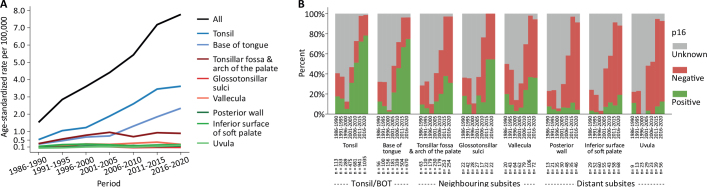
Age-standardised incidence rates, standardised to the 2020 Danish population age distribution (A) and frequency of tumour p16-status (positive, negative and unknown) in OPSCC by subsite (B) in 5-year periods from 1986 to 2020, *N* = 8,462.

A total number of 8,462 patients with OPSCC and known subsite specification were diagnosed in Denmark from 1986 to 2020, Figure 1: upper blue box. Overall, the age-standardised incidence rate of OPSCC increased 5-fold from 1.5 in 1986–1990 to 7.8 in 2016–2020, Figure 2A and Supplementary Table 2. Breaking down the incidence rates by subsite reveals that tonsil and BOT tumours were responsible for the main part of the observed increase in OPSCC incidence over time with a 6.9- and 8.6-fold increase, respectively. Tumours arising in the tonsillar fossa, arch of the palate and vallecula increased around 3-fold, whereas the incidence of tumours arising in any of the remaining subsites was stable over time, Figure 2A and Supplementary Table 2. Tumour p16-status was known in 5,924 (70%) cases, with a significant increase in the proportion with known p16-status over time, Figure 2B. Notably, the interval 1996–2000 is characterised by having very few p16-measurements, yielding a statistically uncertain sample, thus less reliable estimates from this period.

The most pronounced development in viral association during the study period was observed for tonsil/BOT tumours, showing a marked rise in the frequency of p16-positivity over time, from around 10% in the beginning of the study period to reach approximately 75% in 2016–2020, Figure 2B. Tumours of neighbouring subsites constituted 23% (*N* = 1,954) of the total population, and the proportion of p16-positive tumours increased from 5 to 10% early in the study period to 30–50% in 2016–2020. On the other hand, tumours originating in distant subsites were much less frequent throughout the study period (9%, *N* = 785), and the proportion of p16-positive tumours remained low and stable over time (5–15%), Figure 2B. Supplementary Figure 1 demonstrates the frequency of tumour p16-status by subsite in 5-year periods from 1986 to 2020 amongst tumours with known p16-status only, *N* = 5,924.

### Outcome

Table 1 shows patient and tumour characteristics stratified by oropharyngeal subsite and p16-status in the cohort of patients treated with curative intent, *N* = 3,387. Overall, as also illustrated in Supplementary Table 3, patients with p16-positive tumours were younger (61 vs. 64), more likely to be male (78% vs. 69%) and never smokers (32% vs. 3%), had smaller primary tumours (≤T2: 73% vs. 55%), more extensive lymphnode involvement (N+: 88% vs. 71%) and more stage III–IV disease (92% vs. 80%), compared to the p16-negative subgroup. Moreover, more p16-positive patients received concurrent chemotherapy (69% vs. 43%). As also evident from Figure 2, a significant subsite variation in the frequency of tumour p16-expression was observed, as only 1% (*N* = 24) of the p16-positive tumours were found to arise in distant subsites. In the p16-positive distant subsites, group median age was 66 years; there was a high proportion of heavy smokers and more T3/T4 tumours (59%) and N0 disease (46%); and fewer patients received CRT (38%) compared to the p16-positive tonsil/BOT and neighbouring subsites groups, Table 1. However, in comparison with the p16-negative tumours in the distant subsites group, no differences were observed in terms of age, heavy smoking, T3/T4 and N0 or frequency of concurrent chemotherapy although the small number of patients did not allow for a statistical comparison, Table 1.

**Table 1:** Patient and tumour characteristics stratified by oropharyngeal subsite and p16-status in the cohort of patients treated with curative intent, *N* = 3,387.

Characteristic	Tonsil/BOT *N* = 2623		Neighbouring subsites *N* = 568		Distant subsites *N* = 196	
	p16 positive *n* = 2053 (78%)	p16 negative *n* = 570 (22%)	p16 positive *n* = 235 (41%)	p16 negative *n* = 333 (59%)	p16 positive *n* = 24 (12%)	p16 negative *n* = 172 (88%)
**Number**	2053 (100%)	570 (100%)	235 (100%)	333 (100%)	24 (100%)	172 (100%)
**Sex**						
Male	1591 (77%)	405 (71%)	183 (78%)	226 (68%)	21 (88%)	112 (65%)
Female	462 (23%)	165 (29%)	52 (22%)	107 (32%)	3 (12%)	60 (35%)
**Age (years)**						
Median (Min, Max)	61 (32, 91)	63 (38, 86)	61 (40, 88)	63 (42, 84)	66 (52, 79)	64 (43, 81)
**T**						
T1	598 (29%)	84 (15%)	36 (15%)	48 (14%)	5 (21%)	40 (23%)
T2	918 (45%)	204 (36%)	114 (49%)	141 (42%)	5 (21%)	79 (46%)
T3	366 (18%)	163 (29%)	60 (26%)	85 (26%)	10 (42%)	44 (26%)
T4	171 (8%)	119 (21%)	25 (11%)	59 (18%)	4 (17%)	9 (5%)
**N**						
N0	216 (11%)	120 (21%)	45 (19%)	112 (34%)	11 (46%)	79 (46%)
N1	740 (36%)	95 (17%)	62 (26%)	46 (14%)	6 (25%)	27 (16%)
N2	1014 (49%)	317 (56%)	120 (51%)	164 (49%)	7 (29%)	60 (35%)
N3	83 (4%)	38 (7%)	8 (3%)	11 (3%)	0 (0%)	6 (3%)
**Stage**						
Stage 1	32 (2%)	14 (2%)	6 (3%)	20 (6%)	3 (12%)	16 (9%)
Stage 2	102 (5%)	57 (10%)	31 (13%)	66 (20%)	3 (12%)	40 (23%)
Stage 3	742 (36%)	107 (19%)	58 (25%)	54 (16%)	10 (42%)	45 (26%)
Stage 4	1177 (57%)	392 (69%)	140 (60%)	193 (58%)	8 (33%)	71 (41%)
**Smoking**						
Never	669 (33%)	21 (4%)	71 (30%)	5 (2%)	2 (8%)	1 (1%)
Former	963 (47%)	172 (30%)	97 (41%)	89 (27%)	11 (46%)	49 (28%)
Current	418 (20%)	369 (65%)	66 (28%)	239 (72%)	11 (46%)	122 (71%)
Unknown	3 (0%)	8 (1%)	1 (0%)	0 (0%)	0 (0%)	0 (0%)
**Smoking, packyears (PY)**						
Median (Min, Max)	15 (0, 165)	43 (0, 171)	16 (0, 138)	42 (0, 162)	44 (0, 64)	46 (0, 150)
< 30 PY	1338 (65%)	142 (25%)	140 (60%)	77 (23%)	8 (33%)	36 (21%)
≥ 30 PY	685 (33%)	416 (73%)	93 (40%)	255 (77%)	16 (67%)	135 (78%)
Unknown	30 (1%)	12 (2%)	2 (1%)	1 (0%)	0 (0%)	1 (1%)
**Primary treatment**						
ART	641 (31%)	311 (55%)	84 (36%)	216 (65%)	15 (62%)	119 (69%)
ART+C	1412 (69%)	259 (45%)	151 (64%)	117 (35%)	9 (38%)	53 (31%)
**Vital status**						
Median follow-up, years (95%CI)	7.6 (7.4–7.9)	8.5 (7.8–9.4)	8.6 (8.2–9.4)	8.1 (7.6–8.8)	6.7 (4.6–9.2)	8.6 (7.6–9.5)
Alive at 5-year follow-up	1324 (64%)	200 (35%)	141 (60%)	122 (37%)	7 (29%)	76 (44%)
Dead before 5-year follow-up	359 (17%)	336 (59%)	52 (22%)	189 (57%)	13 (54%)	82 (48%)
Alive but follow-up < 5 years	370 (18%)	34 (6%)	42 (18%)	22 (7%)	4 (17%)	14 (8%)
Lost before 5-year follow-up	0 (0%)	0 (0%)	0 (0%)	0 (0%)	0 (0%)	0 (0%)

ART: Accelerated radiotherapy; ART+C: Accelerated radiotherapy with concurrent weekly cisplatin.

**Figure 3:**
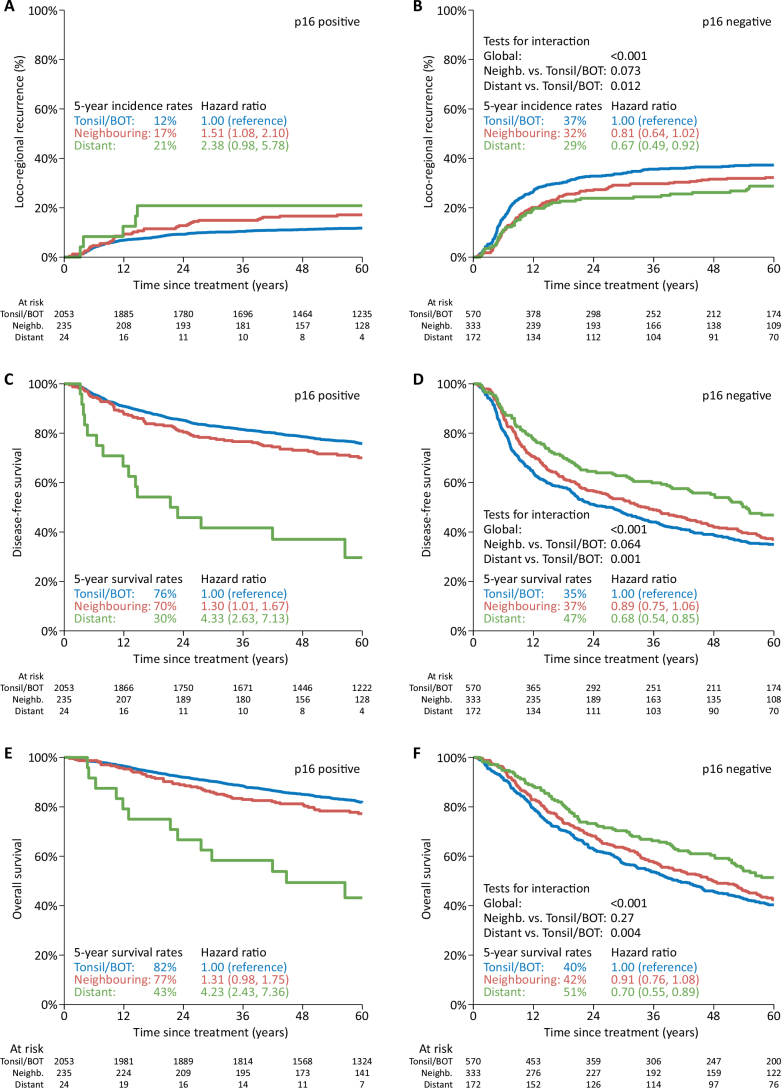
Actuarial estimated loco-regional recurrence (A, B), disease-free survival (C, D) and overall survival (E, F) by oropharyngeal subsite and stratified by p16-status. Tests for interaction evaluate the null hypothesis that the association between p16 status and outcome does not differ across tumour subsites. *N* = 3,387.

The median follow-up time in the cohort treated with curative intent was 7.9 years (7.7–8.1), Table 1 and Supplementary Table 3. The prognostic impact of p16-status stratified by subsite is shown in Supplementary Figures 2, 3 and 4, demonstrating the 5-year risk of loco-regional recurrence and probability of DFS and OS, respectively. Tumour p16-positivity was associated with a statistically significant improvement in all outcomes for all subsites grouped together and for tonsil/BOT and neighbouring subsites, compared with p16-negative disease. However, in tumours arising in distant subsites, no significant impact of p16-status in either endpoint could be detected. Figure 3 shows the actuarial estimated loco-regional recurrence (A, B), DFS (C, D) and OS (E, F) by oropharyngeal subsite and stratified p16-status, respectively, 5 years after completion of treatment. For all endpoints, a test for interaction was performed to evaluate the null hypothesis that the association between p16-status and outcome does not differ across tumour subsites. The global p-values indicate that when all three subsites are grouped together, the null hypothesis is rejected (*p* < 0.001), meaning that there is a statistically significant difference in the prognostic impact of p16-status (estimated by HR) depending on subsite. This was found for all three endpoints examined. An individual comparison of the subsites using tonsil/BOT as reference group showed statistically significant poorer outcomes in the distant subsite group: LRC: *p* = 0.012, DFS: *p* = 0.01 and OS: *p* = 0.004, respectively. No statistically significant differences between tonsil/BOT and neighbouring subsites could be detected. Due to the disproportionate distribution of patients in the respective subsite groups and consequent limited statistical power, no multivariable analysis was performed.

## Discussion and conclusion

### Incidence

Based on this complete nationwide cohort of 8,462 OPSCC with known subsite specification prospectively collected over a period of 35-years, we show that Denmark is a high-incidence area of HPV-related OPSCC. We also demonstrate significant oropharyngeal subsite-specific differences both regarding the age-standardised incidence rates and the proportion of p16-positive tumours. The main part of the OPSCC incidence-rise in Denmark from 1986 to 2020 is explained by the sharp increase in tumours of the tonsil/BOT, with a smaller contribution from tumours arising in neighbouring subsites. As the proportion of HPV-related p16-positivity in tonsil/BOT and neighbouring subsites tumours increased markedly in the same period, the overall development in OPSCC incidence can be ascribed to the frequent occurrence of these particular tumours arising in or near the lymphoepithelial tissue. This is supported by the observation that neither the incidence rates nor the proportion of p16-positivity in OPSCC arising in distant subsites changed over time. Importantly, as the proportion of tumours with known p16-status in our cohort gradually increases over time to constitute ~95% at the end of the study period, the most robust estimates of the impact p16-expression and the derived consequences of viral presence are found in these recent intervals.

### Outcome

In addition to the epidemiological subsite differences, our outcome analysis restricted to patients treated with curatively intended (chemo)radiotherapy showed significant differences in the prognostic impact of p16-expression depending on the subsite. The potent impact of p16-positivity was confirmed in tonsil/BOT and tumours arising in neighbouring subsites, whereas no apparent impact in distant subsite tumours was seen. Similar results were recently published by Marklund et al, although in their study, all non-tonsil/BOT tumours were grouped as ‘other’, and no distinguishment between neighbouring and distant subsite tumours was available [17]. Our findings indicate that neighbouring subsite tumours, due to the anatomical proximity with the lymphoid tissue, probably represent a mixture of biology combined with potential ‘anatomical misclassification’ when the origin of the biopsy is indicated by the surgeon. However, in distant subsite tumours, the low proportion of p16-expression and the lack of prognostic impact are striking and comparable to what has been observed for non-oropharyngeal HNSCC [12], indicating that these tumours are probably characterised by a truly different biology, compared to tonsil/BOT tumours.

The current differences in outcome amongst the subsites are likely to be more expressed than the 35-years average data. This is due to a combination of relatively more p16-positive tumours observed especially in the tonsil/BOT cohort together with a substantial improved treatment outcome due to the introduction of various radiobiological-based treatment modifications (hypoxic modification, acceleration and chemo-radiotherapy) [23]. All this has gradually been implemented in the Dahanca guidelines, and the described outcome data from the period 2011 to 2020 (Supplementary Figures 2–4) consequently describe the expected current outcome and variation as a function of subsite.

Besides p16, different diagnostic assays exist to detect HPV, including DNA and RNA in-situ hybridisation, DNA and RNA PCR, HPV DNA sequencing and algorithms combining more of these methods. Which method most reliably detects the ‘true’ HPV attributable fraction (HPV-AF) in OPSCC continues to be of debate in the medical community [7]. Mehanna et al published a multinational study, pooling individual patient data and found discordance between p16 IHC and HPV DNA/RNA (p16+/HPV- or p16-/HPV+) in approximately 10% of patients [16]. Importantly, they demonstrated that the higher the HPV-AF, the lower the number of discordant cases. In addition, the frequency of discordant cases was highest in subsites ‘outside the tonsil/BOT’, which were not otherwise specified anatomically. These findings suggest a less robust correlation between HPV and p16-expression in tumours arising ‘outside the tonsil/BOT’, which probably contributes to the overall lack of prognostic impact of p16-expression in those subsites. Of note, amongst tumours with discordance, patients with p16-/HPV+ tumours had poorer outcome than the p16+/HPV- subgroup [7], which was also demonstrated in a meta-analysis evaluating the clinical relevance of HPVDNA-positivity and p16-expression in squamous cell carcinomas of all head and neck sites [24].

The TNM8 classification, introducing separate staging criteria based on tumour p16-expression, was developed and validated based on the ICON-S staging system derived from a multicentre cohort, including all OPSCC without consideration of potential subsite variation [15]. As shown, the prevalence of OPSCC arising in distant subsites is low, especially the p16-positive subgroup. Consequently, our population-based results constitute a valuable contribution to the understanding of these important subsite differences, due to the large number of patients included in the cohort along with uniform treatment strategies and complete follow-up. Moreover, as almost all OPSCC patients (98%) in the ICON-S cohort were treated with primary (chemo)radiotherapy, comparable to the treatment applied in our study, this yields a high agreement between our study population used for outcome analysis and the ICON-S cohort, adding to the strength of our findings. As the number of patients with p16-positive distant subsite tumours in our study were too low to allow for regular multivariable analysis, it cannot be ruled out that there is a skewness in terms of other prognostic factors between the subgroups with potential significant influence on outcome, which limits the ability to account for confounding variables. However, the global test for interaction as well as the comparison between tonsil/BOT and distant subsite tumours were statistically significant, supporting that in OPSCC arising in distant subsites, there is no prognostic impact of p16-status. Consequently, it should be considered whether these tumours in terms of biology and treatment response have a higher resemblance to non-OPSCC and, therefore, preferably should not be classified in TNM according to p16-status.

## Conclusion

In conclusion, we demonstrate that the fivefold increase in the age-standardised incidence of OPSCC in Denmark from 1986 to 2020 is ascribable to the rise in HPV-related p16-positive tumours of tonsil/BOT and neighbouring subsites only. The prognostic impact of p16-status was shown to differ across tumour subsites with the strongest association found in tonsil/BOT tumours, a diminishing but still significant impact in tumours arising in neighbouring subsites and no significant impact in tumours arising in distant subsites. This suggests that grouping all p16-positive OPSCC as one entity for staging and prognostication, as currently done in TNM8, is too simple as it does not accurately depict the differences in biology and the consequent treatment response of the tumours. Consequently, our findings are likely to have implications for a refinement of the OPSCC TNM-classification and planning of future treatment strategies for patients with OPSCC.

## Supplementary Material



## Data Availability

Anonymised data sharing will be possible upon reasonable request to the corresponding author and when in agreement with the DAHANCA-group and the Danish legislation.
