# Supplementary material for: Subsite variation of HPV-related p16-expression in oropharynx cancer: Incidence and prognostic impact in a population-based DAHANCA cohort 1986–2020
Source: Acta Oncol. 2025 Aug 14;64:44027. doi: 10.2340/1651-226X.2025.44027 (PMC12371743; doi:10.2340/1651-226X.2025.44027)

Supplementary material has been published as submitted. It has not been copyedited, or typeset by Acta Oncologica

Supplementary Material

Subsite variation of HPV-related p16-expression in oropharynx cancer: Incidence and prognostic impact in a population based DAHANCA cohort 1986-2020

Pernille Lassen, Jan Alsner, Hanne Primdahl, Caroline C Plaschke, Christian Maare,

Jørgen Johansen, Maria Andersen, Mohamad Farhadi, Jens Overgaard

Supplementary Table 1

Disease classification 1

Supplementary Table 2

Age-standardized incidence rates, all Danish OPC patients 1986-2020 2

Supplementary Table 3

Patient and tumour characteristics stratified by p16-status 3

Supplementary Figure 1

Frequency of tumour p16-status (positive vs. negative) by oropharyngeal subsite 4

Supplementary Figure 2

Loco-regional recurrence by p16-status stratified by oropharyngeal subsite 5

Supplementary Figure 3

Disease-free survival by p16-status stratified by oropharyngeal subsite 6

Supplementary Figure 4

Overall survival by p16-status stratified by oropharyngeal subsite 7

Supplementary Table 1. Disease classification used in the study.

T-classification	N-classification	M-classification	Stage
T1: T1	N0: N0	M0: M0	Stage I: T1N0
T2: T2	N1: N1	M1: M1	Stage II: T2N0
T3: T3	N2: N2a+N2b+N2c		Stage III: T3N0+T1/T2/T3N1
T4: T4a+T4b	N3: N3a+N3b		Stage IV: T1/T2/T3 N2/N3 + T4 Any N + M1 Any T Any N

Supplementary Table 2. Age-standardized incidence rates, standardized to the 2020 Danish population age distribution. Includes all Danish OPC patients 1986-2020, N=8,462.

Incidence year	Tonsil	Base of tongue	Tonsillar fossa & arch of the palate	Glossotonsillar sulci	Vallecula	Posterior wall	Inferior surface of the soft palate	Uvula
1986-1990	0,527	0,269	0,296	0,101	0,091	0,064	0,138	0,041
1991-1995	1,046	0,461	0,559	0,196	0,196	0,096	0,238	0,057
1996-2000	1,225	0,676	0,783	0,115	0,278	0,154	0,266	0,105
2001-2005	1,890	0,740	0,949	0,114	0,252	0,119	0,226	0,123
2006-2010	2,598	1,307	0,698	0,064	0,318	0,186	0,167	0,094
2011-2015	3,454	1,846	0,928	0,079	0,381	0,128	0,219	0,146
2016-2020	3,618	2,341	0,889	0,078	0,253	0,163	0,237	0,197

Supplementary Table 3. Patient and tumour characteristics stratified by p16-status in the cohort of patients treated with curative intent, N=3,387.

	All	p16 positive	p16 negative	p-value
**Number**	3,387 (100%)	2312 (68%)	1075 (32%)	
**Sex**				
Male	2538 (75%)	1795 (78%)	743 (69%)	<0.0001
Female	849 (25%)	517 (22%)	332 (31%)	
**Age (years)**				
Median (Min,Max)	62 (32,91)	61 (32,91)	64 (38,86)	<0.0001
**Subsite**				
Tonsil/BOT	2623 (77%)	2053 (89%)	570 (53%)	<0.0001
Neighbouring subsites	568 (17%)	235 (10%)	333 (31%)	
Distant subsites	196 (6%)	24 (1%)	172 (16%)	
**T**				
T1	811 (24%)	639 (28%)	172 (16%)	<0.0001
T2	1461 (43%)	1037 (45%)	424 (39%)	
T3	728 (21%)	436 (19%)	292 (27%)	
T4	387 (11%)	200 (9%)	187 (17%)	
**N**				
N0	583 (17%)	272 (12%)	311 (29%)	<0.0001
N1	976 (29%)	808 (35%)	168 (16%)	
N2	1682 (50%)	1141 (49%)	541 (50%)	
N3	146 (4%)	91 (4%)	55 (5%)	
**Stage**				
Stage 1	91 (3%)	41 (2%)	50 (5%)	<0.0001
Stage 2	299 (9%)	136 (6%)	163 (15%)	
Stage 3	1016 (30%)	810 (35%)	206 (19%)	
Stage 4	1981 (58%)	1325 (57%)	656 (61%)	
**Smoking**				
Never	769 (23%)	742 (32%)	27 (3%)	<0.0001
Former	1381 (41%)	1071 (46%)	310 (29%)	
Current	1225 (36%)	495 (21%)	730 (68%)	
Unknown	12 (0%)	4 (0%)	8 (1%)	
**Smoking, packyears (PY)**				
Median (Min,Max)	27 (0,171)	15 (0,165)	43 (0,171)	<0.0001
<30 PY	1741 (51%)	1486 (64%)	255 (24%)	<0.0001
≥30 PY	1600 (47%)	794 (34%)	806 (75%)	
Unknown	46 (1%)	32 (1%)	14 (1%)	
**Primary treatment***				
ART	1386 (41%)	740 (32%)	646 (60%)	<0.0001
ART+C	2001 (59%)	1572 (68%)	429 (40%)	
**Vital status**				
Median follow-up, years (95%CI)	7.9 (7.7-8.1)	7.8 (7.5-8.0)	8.4 (7.9-9.0)	
Alive at 5-year follow-up	1870 (55%)	1472 (64%)	398 (37%)	
Dead before 5-year follow-up	1031 (30%)	424 (18%)	607 (56%)	
Alive but follow-up <5 years	486 (14%)	416 (18%)	70 (7%)	
Lost before 5-year follow-up	0 (0%)	0 (0%)	0 (0%)	

*ART: Accelerated radiotherapy, ART+C: Accelerated radiotherapy with concurrent weekly cisplatin

Supplementary Figure 1. Frequency of tumour p16-status (positive vs. negative) in OPSCC by subsite in 5-year periods from 1986-2020, N= 5,924 (2,538 p16 unknown are excluded). Numbers above bars indicate number of p16 positive / total number of p16 positive and negative.

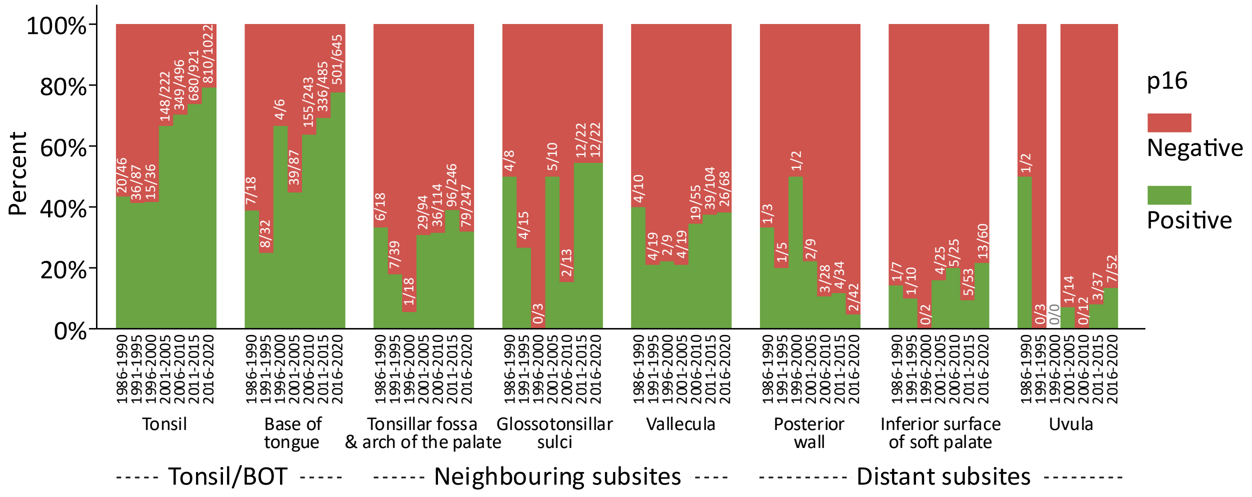

Supplementary Figure 2. Actuarial estimated loco-regional recurrence by p16-status stratified by oropharyngeal subsite – Tonsil/BOT (A), Neighbouring subsites (B), Distant subsites (C) and All subsites (D). N=3,387.

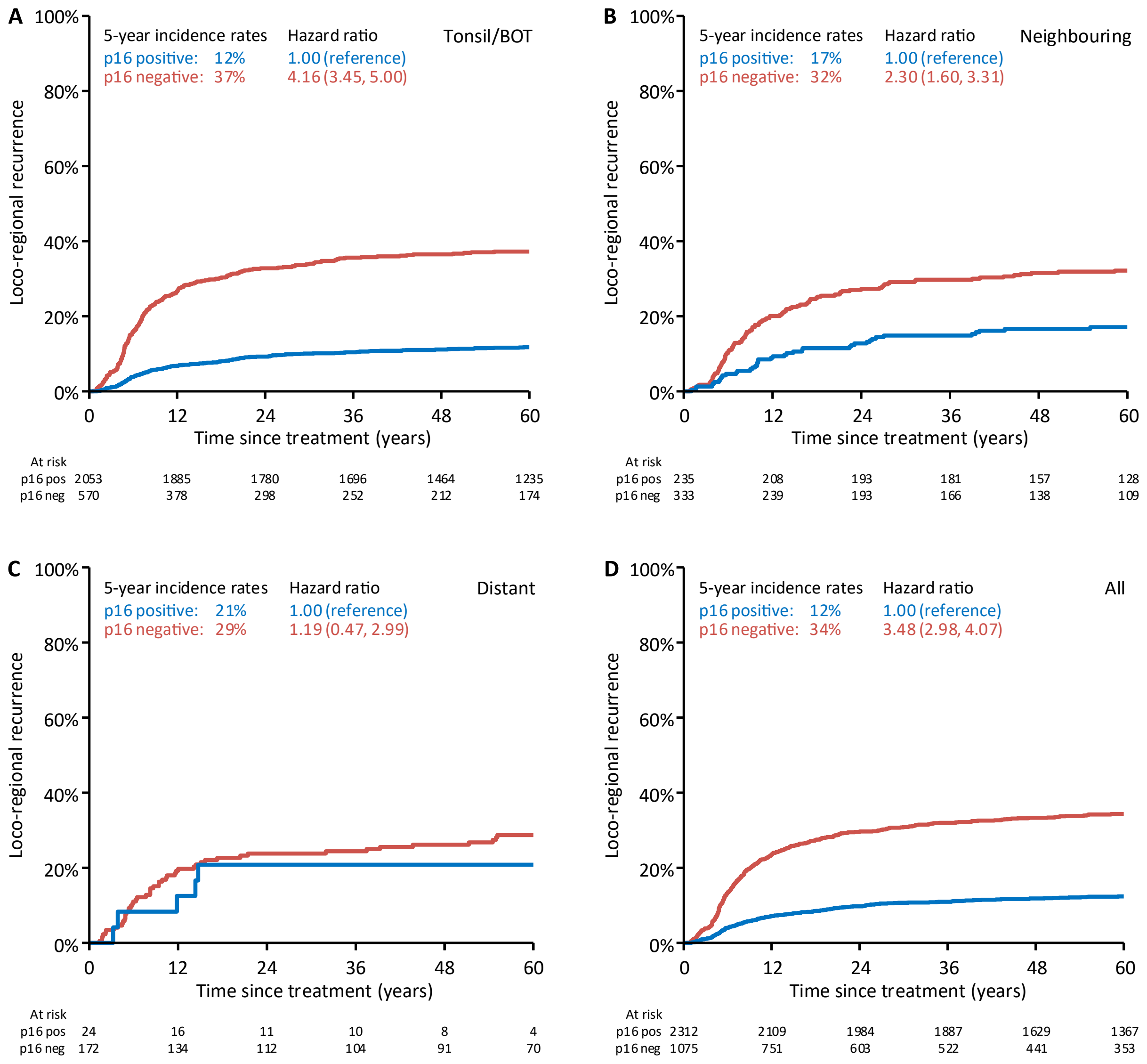

Supplementary Figure 3. Actuarial estimated disease-free survival by p16-status stratified by oropharyngeal subsite – Tonsil/BOT (A), Neighbouring subsites (B), Distant subsites (C) and All subsites (D). N=3,387.

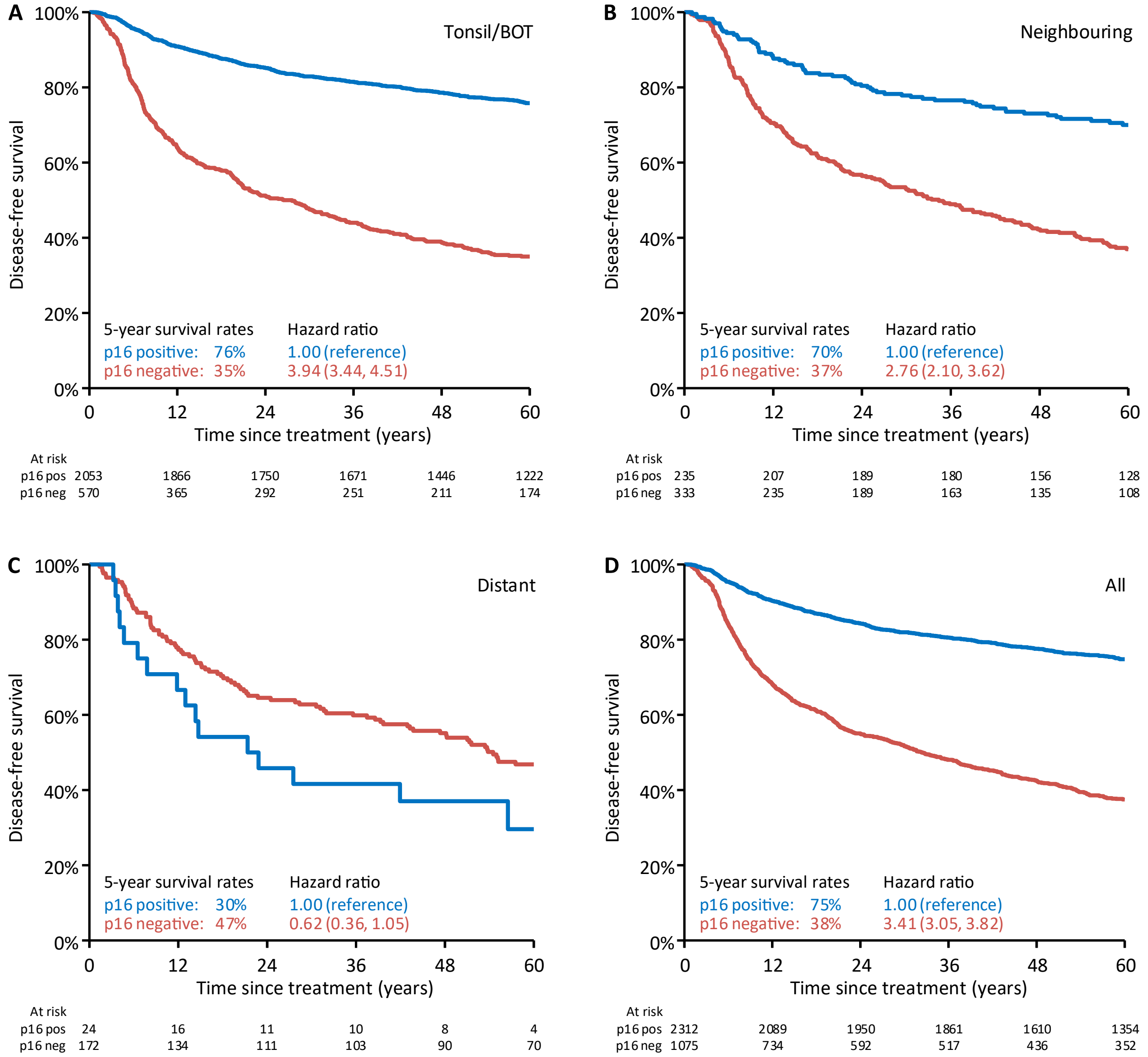

Supplementary Figure 4. Actuarial estimated overall survival by p16-status stratified by oropharyngeal subsite – Tonsil/BOT (A), Neighbouring subsites (B), Distant subsites (C) and All subsites (D). N=3,387.

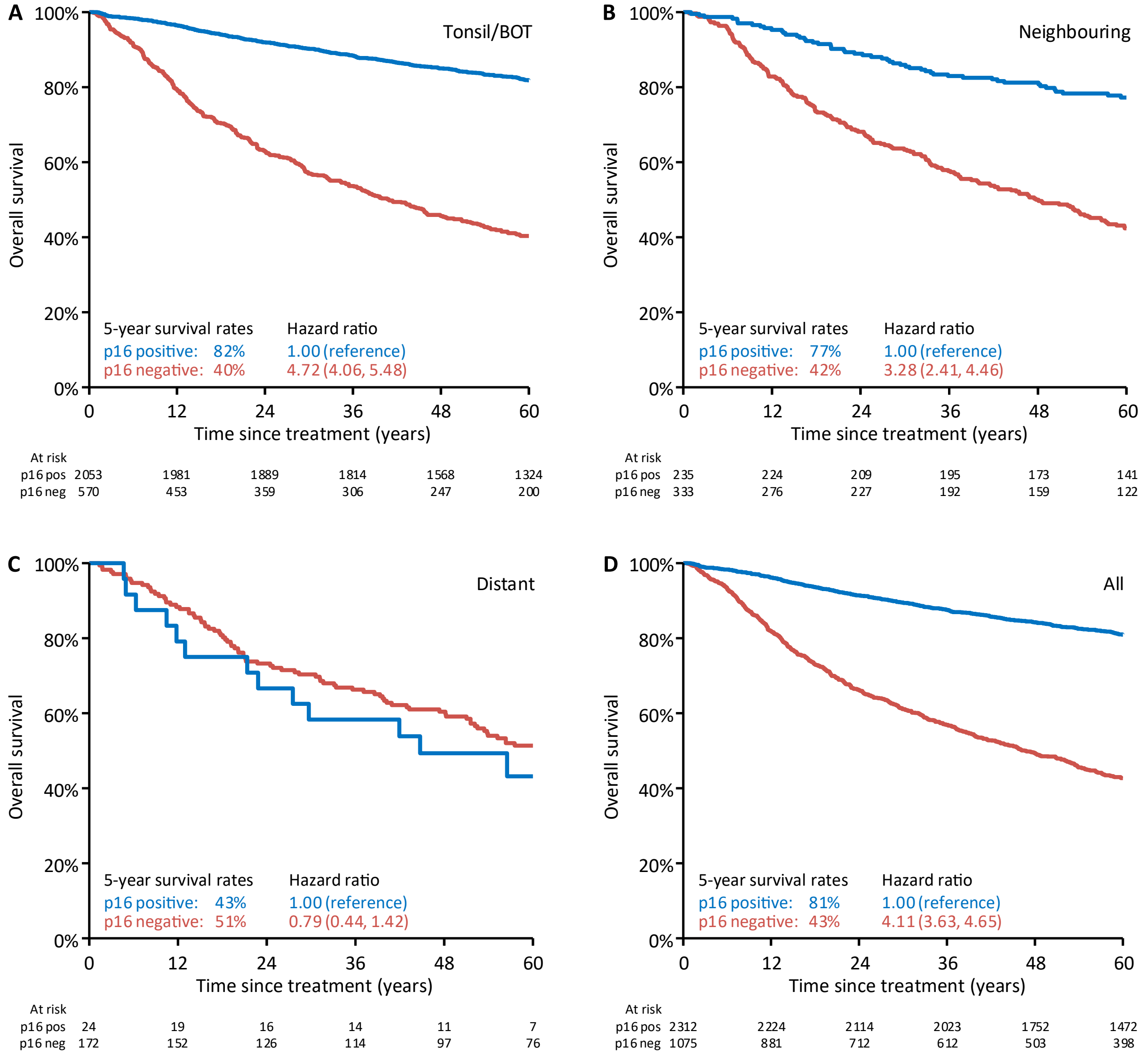

Supplement: Supplementary file 1 [file AO-64-44027-s1.pdf]
